# Experienced entropy drives choice behavior in a boring decision-making task

**DOI:** 10.1038/s41598-022-06861-w

**Published:** 2022-02-24

**Authors:** Johannes P.-H. Seiler, Ohad Dan, Oliver Tüscher, Yonatan Loewenstein, Simon Rumpel

**Affiliations:** 1 Institute of Physiology, Focus Program Translational Neurosciences (FTN), University Medical Center of the Johannes Gutenberg University Mainz, Hanns-Dieter-Hüsch-Weg 19, 55131 Mainz, Germany; 2 Department of Psychiatry and Psychotherapy, University Medical Center of the Johannes Gutenberg University Mainz, Untere Zahlbacher Straße 8, 55131 Mainz, Germany; 3 Department of Comparative Medicine, Yale School of Medicine, New Haven, CT 06520 USA; 4 Leibniz Institute for Resilience Research, Wallstraße 7, 55122 Mainz, Germany; 5 The Alexander Silberman Institute of Life Sciences, Department of Cognitive Sciences, The Federmann Center for the Study of Rationality, The Hebrew University of Jerusalem, 9190401 Jerusalem, Israel

**Keywords:** Decision, Human behaviour

## Abstract

Boredom has been defined as an aversive mental state that is induced by the disability to engage in satisfying activity, most often experienced in monotonous environments. However, current understanding of the situational factors inducing boredom and driving subsequent behavior remains incomplete. Here, we introduce a two-alternative forced-choice task coupled with sensory stimulation of different degrees of monotony. We find that human subjects develop a bias in decision-making, avoiding the more monotonous alternative that is correlated with self-reported state boredom. This finding was replicated in independent laboratory and online experiments and proved to be specific for the induction of boredom rather than curiosity. Furthermore, using theoretical modeling we show that the entropy in the sequence of individually experienced stimuli, a measure of information gain, serves as a major determinant to predict choice behavior in the task. With this, we underline the relevance of boredom for driving behavioral responses that ensure a lasting stream of information to the brain.

## Introduction

Boredom is a human experience intimately familiar to all of us. Defined as “an aversive mental state of wanting, but being unable, to engage in satisfying activity”^1^^(p. 483)^, boredom is encountered in a wide range of daily contexts such as school and workplace^2–6^. Moreover, in clinical contexts, boredom has been linked to wide variety of psychopathologies, including attention deficit hyperactivity disorder (ADHD)^7–9^, depression^10–12^, traumatic brain injury^13^ and various impulse control deficits^14–21^.

Despite its omnipresence, boredom has received significant scientific attention only recently and a systematic study of its cognitive characteristics and neurobiological underpinnings has just begun^22,23^. Based on self-report assessments, boredom has been characterized both as a trait and a state^1,22,24^. *Trait boredom* enfolds the general proneness to become bored in a broad range of environments, whereas s*tate boredom* describes a transient experience in response to a particular situation. Together, predisposed proneness to being bored interacts with the features of a present environment to eventually produce the aversive state of boredom.

Previous work has identified different such situational factors that lead to boredom, converging on two main independent factors: first, a lack of meaning and value in a given situation and, second, a lack of attention due to a mismatch of individual cognitive demands and resources^1,25,26^. In line with this theoretical framework, different experimental approaches have been used in order to induce boredom, comprising monotonous motor tasks^27–29^ and monotonous sensory stimulation^27,30,31^. These approaches however, put their emphasis on the induction and measurement of boredom experience, but did not allow to analyze and gradually manipulate the environmental features causing boredom. An investigation and description of these features in a parametric manner thus remains open. Intuitively, the high degree of predictability in a monotonous environment emerges as a candidate.

In information theory, *entropy* quantifies the predictability of a sequence of inputs in units of information^32^. For example, the visual entropy in a monotonous video sequence showing drying paint would be low because a present frame is highly predictive of the next one (the next frame contains very little new information). In contrast, the visual entropy of a thrilling cinematic movie is potentially much greater since the next frame could reveal a lot of new information. If environmental monotony is indeed a major determinant in boredom, then, from an information-theory perspective, the optimal strategy to alleviate boredom would be to avoid incoming stimuli with low entropy. Here we hypothesize, that low environmental entropy relates to human boredom experience and that the entropy of perceived stimulus sequences acts as a driving force in choice behavior.

We present a paradigm that both elicits boredom under conditions of controlled and scalable environmental monotony while at the same time providing a parametric readout of boredom-related behavior: In a simple psychophysical repeated choice task we offered two alternatives that were coupled with different degrees of repetitive sensory stimulation, and observed that individuals bias their choices as to avoid monotony. By testing the link of this bias to visual analog and established self-report ratings of boredom, we found a robust induction of boredom during the task as well as a positive correlation between monotony avoidance and state boredom. Furthermore, the task experience matched with individuals’ self-reported affect and arousal of imagined boredom experience, while being clearly distinct from curiosity. Furthermore, as the task design allowed a straightforward quantification of a subject’s experience that results in boredom, we developed a theoretical framework to model the dynamics of individual choices in the task and identified empirical entropy as a key driver of boredom-related choice.

## Results

### The Boredom Choice Task

**Figure 1:**
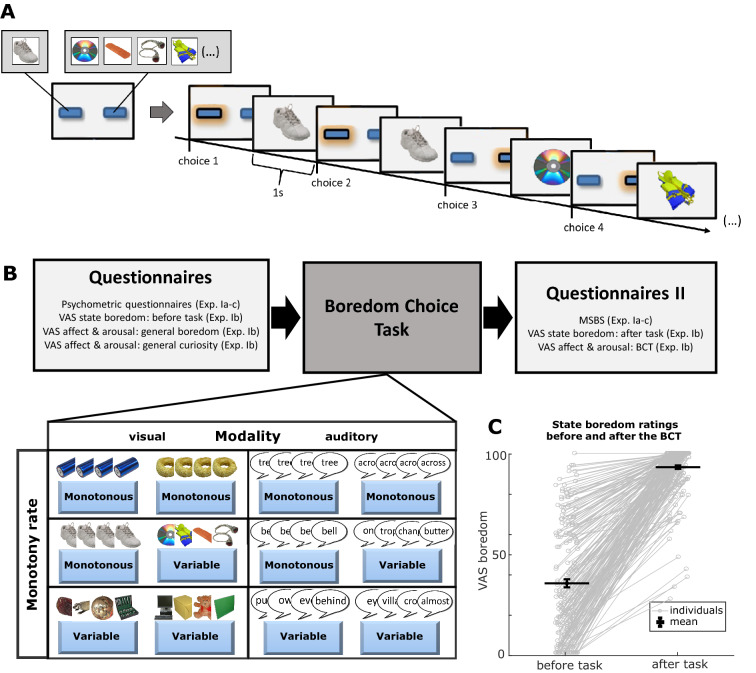
Concept of the *Boredom Choice Task*: (**A**) Trial structure of the paradigm and example sequence of choices and stimulus presentations in the *monotonous vs. variable* condition, where the monotonous alternative in this example is located left. The shaded buttons represent the currently chosen options. (**B**) Schematic of the procedure, illustrating six basic task conditions (*monotonous vs. variable, monotonous vs. monotonous, variable vs. variable* in visual and auditory modality). Images are examples from the visual stimulus libraries taken from the Bank of Standardized Stimuli^59,60^. The structure of the experiments involved the BCT and various self-report questionnaires to assess boredom (BPS, MSBS, VAS-B), personality traits and symptoms of mental disorders (GHQ-28, CAARS-S:L, BDI-II, BFI-10, I-8, STAI-Y, BRS) as well as affect and arousal for imagined states of boredom, curiosity and the BCT (VAS-AA). (**C**) Visual analog state boredom ratings (VAS-B) before starting and after completing the Boredom Choice Task. Connected grey circles reflect the ratings of each individual (n = 201 participants). The horizontal bars reflect the average over all subjects and the vertical bars indicate the standard error of the mean. The boredom ratings after the task are significantly higher compared to the prior condition (p < 0.001 in a Wilcoxon signed rank test).

In order to study boredom in the context of a defined task that both induces boredom to a controllable degree and at the same time provides a behavioral readout, we focused on a consensus definition of a well operationalizable key feature of boredom, namely negative affect^1,33–35^. We measured the participants’ degree of avoidance of different sources of sensory stimulation that varied in their level of monotony^1,34^. Participants were instructed to perform a repeated two-alternative forced choice task, in which each alternative was coupled with the presentation of different sensory stimuli (Fig. 1A). Importantly, the alternatives were not associated with any form of reward or punishment. The stimuli linked with each of the alternatives were drawn from stimulus libraries of varying sizes, allowing the operationalization of different levels of entropy yielding a direct measurement of how they drive behavior. When investigating the visual modality, libraries consisted of images of neutral objects, whereas for the auditory modality we used recordings of single spoken words with neutral meaning (see “Materials and methods”). In the following, we refer to this paradigm as the *Boredom Choice Task* (BCT).

To link behavior in the task to established measures of boredom, subjects also completed diverse questionnaires and visual analog scale ratings (VAS asking “Please rate on the slider below how bored you feel at this moment.”, see Additional Information) before and after performing multiple cycles of the BCT (Fig. 1B). We observed a substantial increase in VAS ratings of subjectively perceived boredom in all subjects, demonstrating a robust induction of boredom during the task, independent of the participants’ particular choices (mean ± SEM for VAS-B before task: 35% ± 2%, VAS-B after task: 91% ± 1%, n = 201 participants, one-tailed Wilcoxon signed rank test: p < 0.001; Fig. 1C).

In the following results section, we present two main sets of experiments. In Experiments Ia–c, we devised and used the BCT with a monotonous and a highly variable alternative to initially assess the effect of a high difference in monotony on choice behavior, to replicate our observations in the visual and auditory modality and to validate our findings in laboratory and online settings. In Experiment II, we systematically compared alternatives with various degrees of monotony and devised a quantitative model, linking the boredom-related choice bias to experienced empirical entropy.

### Monotonous sensory stimulation is aversive

**Figure 2:**
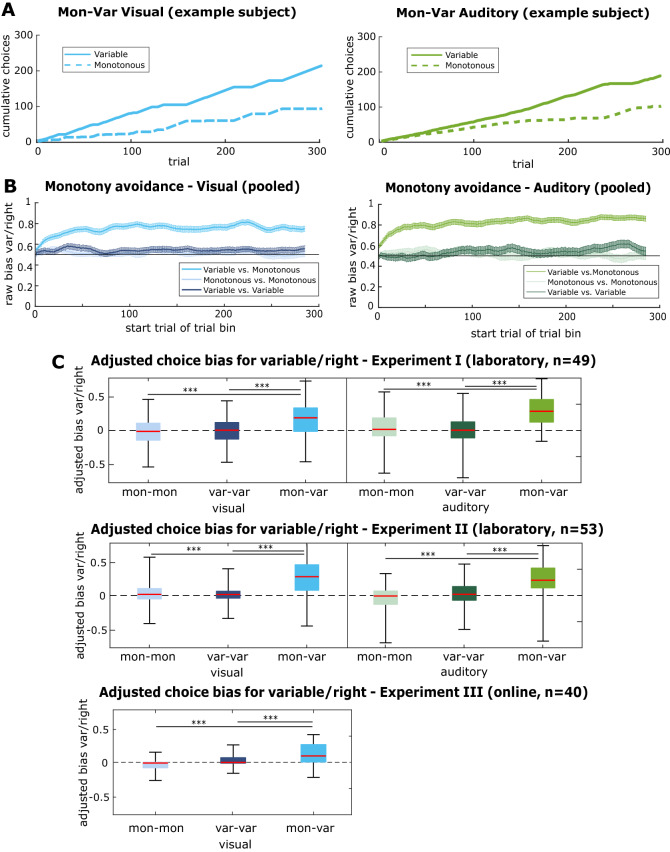
Boredom bias of monotony avoidance in the Boredom Choice Task: (**A**) Choice behavior of one exemplary subject in the visual and auditory *monotonous vs. variable* (*Mon–Var*) BCT cycle. The cumulative number of choices for either alternative is plotted over the respective trial. (**B**) Average *raw boredom bias* of all participants from Experiment Ia-c (n = 142 participants for visual modality, n = 102 participants for auditory modality) over the duration of each task cycle across all conditions. The raw boredom bias is computed in a bin of 15 trials (first bin: trial 1–15) which is then shifted stepwise until the end of the task (last bin: trial 286–300). The vertical bars indicate the standard error of the mean. (**C**) Boxplots with the distributions of the *adjusted boredom bias* for Experiment Ia (n = 49 participants), Ib (n = 53 participants) and Ic (n = 40 participants). The red line indicates the median, the box indicates the upper and lower 25% quantiles and the whiskers indicate the 50% quantiles around the median. Blue colors reflect visual task cycles, whereas green colors represent auditory task cycles. In all experiments the *Mon–Var* distributions were significantly different from a mean of zero (***p < 0.001 in one-sample *t* tests).

In the first set of experiments (Experiments Ia–c), the two alternatives in the BCT were linked to sensory stimulation with either different degrees of monotony (monotonous vs. variable library sizes (*Mon–Var*): auditory 1:300, visual 1:449) or similar degrees of monotony (monotonous vs. monotonous library sizes (*Mon–Mon*): auditory 1:1, visual 1:1; variable vs. variable library sizes (*Var–Var*): auditory 150:150, visual 225:225), serving as controls for non-sensory choice biases^43^. We performed three independent experiments, two under laboratory conditions for which we recruited healthy undergraduate students (Experiment Ia, n = 49 participants, Experiment Ib, n = 53 participants; see Supplementary Table 1). An additional experiment was conducted using an online platform (Amazon Mechanical Turk; Experiment Ic, n = 40 participants). We consistently observed an avoidance bias of the alternative associated with more monotonous stimulation (Fig. 2A, Supplementary Fig. 1) that we refer to as *boredom bias*. When calculating the *raw boredom bias* as the proportion of choices to the variable alternative, we observed that across the population, this bias developed within few tens of trials and reached a plateau of approximately 80% of choices avoiding the monotonous option (Fig. 2B). In addition, individual subjects, however, exhibited idiosyncratic choice biases in favor of one side^36^ (Supplementary Fig. 1). To compensate for these idiosyncratic biases, we calculated an *adjusted boredom bias* that expressed the choice bias detected in the *Mon–Var* condition relative to the choice biases that were measured in the symmetric control conditions (see “Materials and methods”, Supplementary Fig. 2). The adjusted boredom bias (Fig. 2C) further demonstrates that participants exhibited a pronounced and significant avoidance of the monotonous alternative (median ± SD for adjusted choice biases: Experiment Ia: visual: 0.20 ± 0.27, auditory: 0.30 ± 0.24, n = 49 participants; Experiment Ib: visual: *Mon–Var*: 0.29 ± 0.27, auditory: *Mon–Var*: 0.24 ± 0.24, n = 53 participants; Experiment Ic: visual: *Mon–Var*: 0.12 ± 0.20, n = 40 participants; One-sample *t* tests comparing the boredom bias against a mean of zero: p < 0.001 in all conditions).

Consistent with the idea that boredom is a phenomenon spanning multiple sensory modalities, the magnitude of avoidance of the monotonous alternative developed to a similar degree in the BCT cycles involving the visual and the auditory modality (compare Fig. 2B,C left and right). However, avoidance of monotony was slightly less pronounced in the online Experiment (Ic), compared with the laboratory Experiments (Ia and b; median ± SD for adjusted boredom bias in Experiment Ia + b: visual *Mon–Var*: 0.22 ± 0.28, n = 102 participants; Experiment Ic: visual *Mon–Var*: 0.12 ± 0.20, n = 40 participants; one-tailed Wilcoxon ranked sum test p = 0.04), which could be due to less controlled experimental conditions (Supplementary Fig. 3).

### Monotony avoidance is linked to experienced state boredom

**Figure 3:**
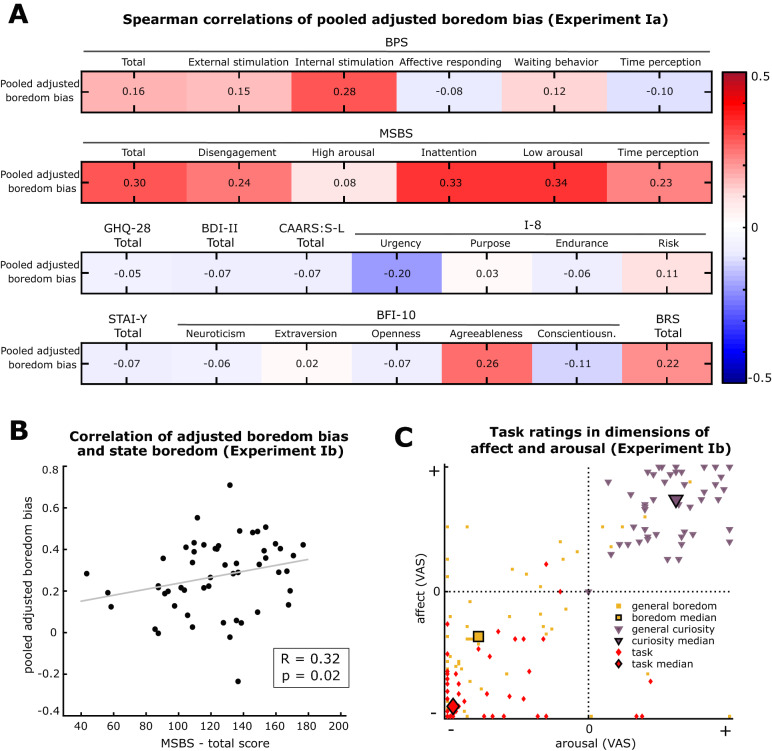
Construct validation of the Boredom Choice Task: (**A**) Exploratory investigation of the Spearman correlations between the pooled *adjusted boredom bias* and the diverse psychometric self-reports (*BPS* Boredom Proneness Scale, *MSBS* Multidimensional State Boredom Scale, *GHQ-28* General Health Questionnaire, *BDI-II* Beck’s Depression Inventory, *CAARS:S-L* Conner’s Adult ADHD Rating Scale, *I-8* Impulsivity Questionnaire, *STAI-Y* State Trait Anxiety Inventory, *BFI-10* Big Five Inventory, *BRS* Brief Resilience Scale). Each correlation is computed over n = 49 participants from Experiment Ia. The color of each cell displays the magnitude of correlation (R-value). (**B**) Specific correlation analysis with the independent data from Experiment Ib: The scatter plot illustrates the relationship between the pooled *adjusted boredom bias* of each participant and the corresponding *MSBS state boredom report* (n = 53 participants; Spearman’s R = 0.32, p = 0.02). The grey line indicates the best linear fit. (**C**) Scatter plot of participants’ visual analog scale (VAS) ratings of affect and arousal for *imagined boredom* (yellow), *imagined curiosity* (violet) and the *BCT experience* (red) (n = 53 participants from Experiment Ib). The large markers indicate the overall median of each condition.

Next, we tested how the behavioral monotony avoidance observed in the BCT relates to self-reported boredom experience. Therefore, we conducted a stepwise correlation analysis. First, we conducted an exploratory correlation analysis with the dataset obtained from Experiment Ia, investigating the relationship between participants’ adjusted boredom bias and various psychometric features that were assessed with self-report questionnaires (Fig. 3A). These questionnaires included standard tests for mental health problems and personality features (GHQ-28, CAARS-S:L, BDI-II, BFI-10, I-8, STAI-Y, BRS) as well as current tests for state boredom (MSBS) and trait boredom (BPS) (see “Materials and methods” for detailed descriptions). In our exploratory analysis, we found appreciable positive correlations of adjusted boredom bias with state boredom, measured by the MSBS (Spearman’s R = 0.30), especially to the MSBS subdimensions of inattention (Spearman’s R = 0.33) and low arousal (Spearman’s R = 0.34), as well as to the internal stimulation subscale of the BPS (Spearman’s R = 0.28). Importantly, monotony avoidance did not noteworthily correlate to self-reported symptoms of mental disorders or other distress factors, suggesting a fairly specific measure for *non-pathological* state boredom in this cohort of healthy subjects.

In a second step, we performed a prospective correlation analysis with the independent dataset from Experiment Ib in order to confirm and replicate our initial observation. This single test of the a priori hypothesis that there is a positive correlation between monotony avoidance bias and experienced state boredom (MSBS sum score), was not impaired by multiple testing bias due to the independence of the datasets (see “Materials and methods”). Here, the correlation between the adjusted boredom bias and self-reported state boredom was again observed on a statistically significant level (Spearman’s R = 0.32, p = 0.02, Fig. 3B). Together, these observations demonstrate that individuals, who report high state boredom, also show a stronger avoidance of the monotonous alternative in the BCT.

### Dissociating curiosity and boredom in the Boredom Choice Task

In principle, classical novelty-seeking related phenomena like *curiosity* could also result in the avoidance of a monotonous alternative compared to a variable alternative. Curiosity, however, in contrast to boredom, is associated with positive affect^37^. Therefore, in another validation step, we tested the linkage of the BCT to curiosity and boredom with respect to the two dimensions of affect and arousal, which are commonly applied to characterize emotions^38–40^ (VAS asking participants “Please rate on the sliders below how happy and aroused you feel in this situation.” for imagined boredom, imagined curiosity and the task experience, see Additional Information). The imagined state of boredom was found to be, on average, rated as aversive and low arousing, whereas imagined curiosity on the other hand was rated with positive valence and high arousal, as expected (Fig. 3C). Importantly, the participants’ experience during the BCT was rated strongly aversive and poorly arousing, thus showing qualitative similarity to the imagined boredom state. This similarity was statistically confirmed through a significant positive correlation between subjects’ boredom and BCT ratings of affect and arousal (Supplementary Table 2). Together, this analysis corroborates the linkage of the behavioral bias and state boredom by showing experiential similarity between task experience and imagined boredom in respect to affect and arousal.

### Measuring the boredom bias over different degrees of monotony

**Figure 4:**
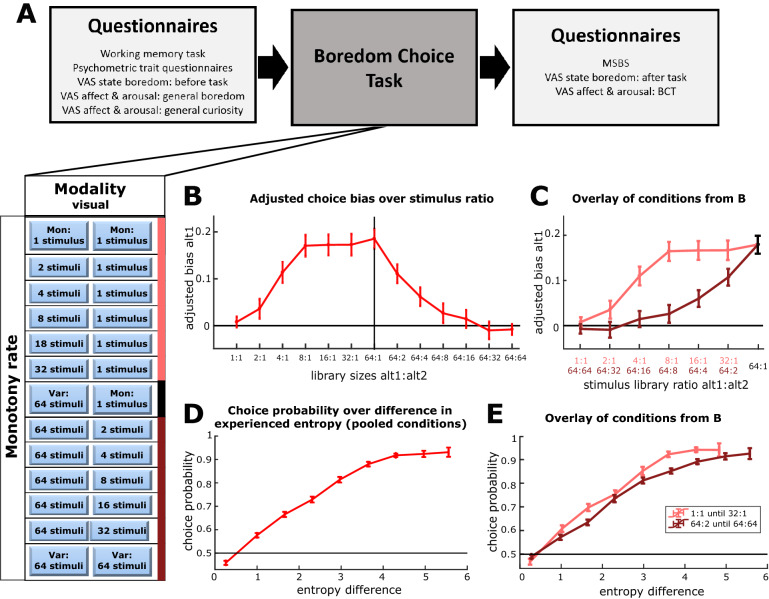
Boredom bias at varied degrees of monotony and its link to empirical entropy: (**A**) Procedure of Experiment II and the corresponding stimulus library ratios of each task cycle. Subjects underwent all 13 conditions in a randomized order. (**B**) The average *adjusted boredom bias* is computed over all subjects from Experiment II (n = 148) for each of the 13 stimulus library pairings. The vertical bars indicate the standard error of the mean. (**C**) The same *adjusted boredom bias* data from B is plotted over the *ratio of stimulus libraries* with both sides of the previous plot (1:1 to 32 and 64:64 to 2) overlaid. Here, both sets of conditions show an incongruent shape, despite equivalent library ratios. (**D**) Average *probability of choice* for one alternative presented over the previously experienced *difference in entropy* for this alternative. Thereby, *entropy difference* and the consecutive *choice probability* is computed for all trials in all BCT cycles of each subject’s (99 choices per 13 cycles resulting in 1287 pairs of data per subject). For negative values of *entropy difference*, the data pairs are inverted, leading to only positive values of *entropy difference*. Next, the data pairs of each subject are sorted into 9 equally spaced bins in the range of [0, 6] according to their *entropy difference* value and *choice probability* is computed over the choice data of each bin. Finally, the binned entropy difference and choice probability are averaged over all 148 subjects, leading to the plotted curve. The bars indicate the standard error of the mean (horizontal bars are vanishingly small). (**E**) In analogy to D, the *entropy difference* and consecutive *choice probability* is computed for each trial, where the data is grouped into two sets of different task conditions that in C showed high divergence (1:1 to 32:1 versus 64:2 to 64:64). In line with the previous procedure, we analyze *entropy difference* and the consecutive choice for each subject in the two sets of conditions (99 trials per 6 cycles resulting in 594 data pairs for each condition set). For negative values of *entropy difference*, the data pairs are inverted, leading to only positive values of *entropy difference*. For each subject the data is sorted into 9 evenly equally spaced bins in the range of [0, 6] according to the experienced *entropy difference* and the *choice probability* is calculated for each bin. The individual data of all 148 participants is then averaged leading to the plotted curves. Different from (**C**), both sets of conditions show a widely congruent relationship between experienced *entropy difference* and consecutive *choice probability*.

The boredom bias of Experiments Ia–c emerged when a fully monotonous alternative, where the same stimulus was presented over and over again, was juxtaposed to a highly variable alternative where the vast majority of individual stimuli were presented only once. In order to dissect how the boredom bias depends on different degrees of monotony between the two alternatives, we performed another experiment under laboratory conditions (Experiment II). Here, participants underwent 13 consecutive BCT cycles (100 trials length, randomized order), in which the alternatives were paired with visual stimulus libraries of varying size (see Fig. 4A). These combinations included libraries with the same relative ratios but different absolute sizes (e.g.: 1:4 and 16:64 stimuli). In conditions with highly different degrees of monotony, we again observed the development of a boredom bias, as in Experiments Ia–c (Supplementary Fig. 4). Furthermore, we observed no significant difference in state boredom ratings compared to the previous experiments, indicating that they were comparable in the extent of boredom they induced (median ± SD for MSBS in Experiment Ia–c: 131 ± 32; MSBS in Experiment II: 130 ± 31; Wilcoxon ranked sum test p = 0.92). Next, we computed the average adjusted boredom bias for each combination of monotony levels (Fig. 4B). We found that the bias increased with the ratio between the number of items in each of the libraries. However, this ratio was not the sole determinant of the boredom bias (Fig. 4C). For example, the adjusted boredom bias in the 8:1 experiment was substantially and significantly larger than the adjusted boredom bias in the 64:8 experiment.

One interpretation of this result could have been that this difference is the result of working memory capacity: a participant in a 64:8 experiment may experience the 64-item library as being smaller than its veridical size because she cannot remember that many items. If this is indeed the case, one would predict that the magnitude of the bias in favor of the larger library would increase with working memory capacity. However, we did not find such correlation (Supplementary Table 3), indicating that the absolute number of stimuli associated with the offered alternatives is insufficient in explaining the boredom-related bias.

### Differences in empirical entropy determine the boredom bias

In order to understand the relationship between various levels of monotony and the boredom-related choice bias, we next considered the distribution of stimuli that were actually *experienced* by the participants throughout the task, rather than the pre-set statistics of the different task conditions. Deviations from the experimenter’s pre-set statistics and the subject’s experienced stimulation arise from the specific sampling of the stimulus libraries, which in turn can impact on the sampling strategy in future choices. This is particularly relevant for the choices made during the first phase in each experimental cycle, when the sampling of larger stimulus libraries is incomplete.

We hypothesized therefore, that *empirical entropy* could serve as a more accurate correlate of an individual’s experienced state of monotony during the task that respects the actual encounter of stimuli in each BCT cycle. In information theory, entropy is used as a measure of information that is associated with a message^32^. In the framework of our experiments, the *empirical entropy* that is associated with the string of sampled stimuli for a given alternative, describes the level of information that is being conveyed.

We computed the empirical entropy for each trial and each of the task’s alternatives by considering the stimuli that were sampled up to this trial (see “Materials and methods”; Supplementary Figs. 5 and 6A). In order to relate both alternatives to each other, we calculated the difference between their current entropy scores (Supplementary Figs. 5 and 6B). For each BCT cycle of 100 trials length, this analysis resulted in 99 *entropy difference* scores, each associated with a temporally independent consecutive decision for one of the alternatives, a total of 1287 pairs for each subject performing 13 cycles of the BCT. The dependence of the probability of choice on entropy difference was estimated by binning the entropy difference and averaging choices in each bin across the subjects. This resulted in a monotonically increasing and saturating relationship between the difference in empirical entropy and choice probability (Fig. 4D). Interestingly, when computing this function separately for the two sets of conditions 1:1 to 32:1 and 64:64 to 64:2 (Fig. 4C), we found that the two curves overlap (Fig. 4E). This result indicates that empirical entropy can serve a useful, quantitative description of the features of sensory stimulation that are driving boredom-related decision-making.

### Modeling boredom-related decision-making

**Figure 5:**
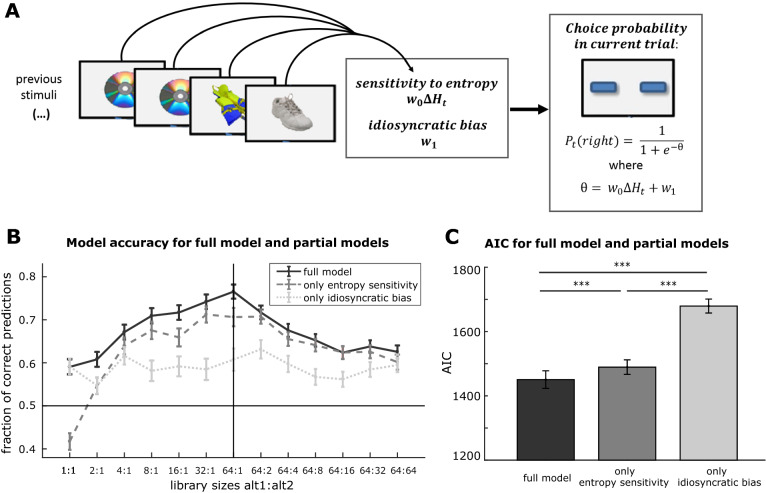
A logistic regression model to explain decision-making in the BCT: (**A**) Schematic explanation of the model and how its parameters are derived from participants’ experience: (i) *sensitivity to entropy* describes how strongly the experienced entropy difference impacts the consecutive choice, and (ii) *idiosyncratic bias* describes a general bias for one alternative. Images are examples from the visual stimulus libraries taken from the *Bank of Standardized Stimuli*^59,60^. (**B**) Average fraction choices that are correctly predicted by the model over the different BCT conditions, where three models are independently fit to the choice data and their predictions are compared (each line presents the average over n = 148 participants, vertical bars indicate the standard error of the mean): (i) the *full model* with two parameters, (ii) a partial model with *only sensitivity to entropy* and (iii) a partial model with *only idiosyncratic bias*. The accuracy of the full model is found to strongly depend on the parameter of entropy sensitivity, where this parameter increases its predictive power as the difference between both alternatives is raised. The idiosyncratic bias on the other hand has a smaller impact on choice predictions that is widely independent from the stimulus libraries of the task. (**C**) Comparison of the model goodness measured as *Akaike’s Information Criterion* (AIC) between the full regression model and partial models with only one parameter (each bar spans the data from n = 148 participants, vertical bars indicate the standard error of the mean). Smaller AIC values indicates a better model. All partial models (only entropy sensitivity and only idiosyncratic bias) show a decreased goodness of fit in comparison to the full model (***: one-tailed Wilcoxon signed rank tests with p < 0.001). Furthermore, the model with only entropy sensitivity performs better than the model with only idiosyncratic bias (***: one-tailed Wilcoxon signed rank test with p < 0.001), indicating that entropy sensitivity is an important determinant of the following choice probability in the BCT.

As non-boredom related factors may also influence the participants’ behavior in the task, we wondered about the relative contribution of empirical entropy and the idiosyncratic choice biases in determining choice behavior. It is well established that in behavioral tasks in which human subjects are forced to choose between two ambiguous alternatives an idiosyncratic bias can develop that substantially influences choice behavior^36,41^. If it was the case that the sensory stimulation would only had a minor impact on the choice behavior during the BCT, it would be expected that idiosyncratic choice biases would play a predominant role. To address this question, we fitted a logistic regression model to the choices of each of the participants (Fig. 5A). According to the model, choice preference is determined by two variables, (i) subjective *sensitivity to entropy* and (ii) *idiosyncratic choice bias*. Each of these variables is associated with a parameter that quantifies the magnitude of its effect on behavior (Fig. 5A).

To quantify the goodness of the model, we used it as a classifier to predict the participants’ individual choices in the different conditions of entropy. To further dissect the impact of the two parameters on model goodness, we compared the fraction of correct predictions of the full model against partial models that were independently fitted to the choice data, including only one of the model parameters (see “Materials and methods”). On average, the full model predicted up to 77% of the choices in one task cycle correctly, where maximal performance was reached in the condition of highly different entropy between alternatives (Fig. 5B). Even in the conditions of equivalent alternatives the model still performed with an average accuracy of approximately 60%. The partial model with only entropy sensitivity showed a similar curve of prediction goodness over task conditions, however with a slightly reduced accuracy. As expected in conditions with a low degree of overall entropy, especially in the 1:1 stimulus condition, this model performed poorly. On the other hand, a model that only utilized the idiosyncratic bias of subjects exhibited a smaller prediction accuracy than the models that involved entropy sensitivity. This prediction goodness of idiosyncratic bias was widely independent from the differences in stimulus libraries. Together, this comparison of the full model with partial models identifies entropy sensitivity as a substantial determinant of behavior in the task.

To corroborate this observation with a standard measure for model comparison, we computed *Akaike’s Information Criterion* (AIC) for the full model and partial models over the full set of choices for each participant (Fig. 5C). Comparing the average AIC for the different models, we found that the full model performed better than both partial models, indicated by a smaller AIC value (mean ± SEM for AIC of full model 1451 ± 27, AIC of model with only entropy sensitivity 1489 ± 23, AIC of model with only idiosyncratic bias 1679 ± 22; one-tailed Wilcoxon signed sum test p < 0.001; n = 148 participants). Comparing the partial models against each other revealed smaller AIC values for the model only involving entropy sensitivity (one-tailed Wilcoxon signed sum test p < 0.001). In sum, this model constitutes a tool to quantitatively compare idiosyncratic and experience-dependent factors that contribute to boredom-related choice behavior in the task at a single trial level, demonstrating a central role of entropy experience for decision-making.

## Discussion

In this study, we present a novel paradigm that induces boredom through sensory stimulation of a particular degree of environmental entropy and simultaneously quantifies behavioral aversion to monotonous sensory stimulation. Through several replications, we found that participants in our Boredom Choice Task avoid alternatives that yield greater stimulus-monotony, and that the extent of this individual aversion is positively correlated with self-reported state boredom. In addition to these findings, we introduce a behavioral model for boredom-driven decision-making and show that participants’ trial-by-trial behavior is, to a large degree, determined by experienced entropy, an information-theory criterion of monotony. Taken together, our findings present boredom in a new light, a natural optimization process for the minimization of experienced information-theory monotony.

### The concept of the Boredom Choice Task

Gauging boredom under controlled environmental circumstances is a necessary condition for its empirical study. While a variety of self-report methods has been developed in order to quantify the subjective experience of boredom^1,23,24,36^, these methods are by nature restricted to humans and measure boredom independent of its environmental causes (e.g. when applying a questionnaire after completing a boring task). The causes of boredom, however, are versatile and range from attentional failures (due to a mismatch of an individual’s cognitive resources and current cognitive demand)^1,25^ over constraint^42^ up to a lack of meaning^25,26^. Therefore, the development of methods that provide a readout of boredom with close linkage to its environmental cause seems auspicious.

Here, we introduce a psychophysical task that induces boredom and simultaneously quantifies boredom-related behavior. This Boredom Choice Task operationalizes sensory monotony as key feature which has been shown to elicit boredom under various experimental conditions^27,29–31^. However, sensory monotony in established boredom-inducing tasks often interfered with intrinsic affective value of the stimulation^30^ or performance-dependent reward^28,31,43^, thus hindering the interpretation of purely sensory effects on boredom. In the Boredom Choice Task we intentionally excluded stimuli with intrinsic affective meaning and performance-dependent rewards in order to maximize the impact of mere sensory features to choice behavior. In addition, as the task requires only a basal level of sustained attention, its behavioral outcome is expected to be widely resistant against individual attentional capabilities. Hereby, we establish an experimental setting in which factors affecting boredom on the levels of meaning and attention^25^ are kept constant and differences in the choice behavior are induced by the two parametrically controlled sources of sensory stimulation. We find that the sensitivity to the information content in the sensory streams, as measured by the choice bias in individual participants, is correlated to their self-reported boredom.

### Validation and limitation of the behavioral boredom measure

The operationalization of boredom in our task is founded on the close reciprocal interplay of boredom and subsequent behavior. In this respect, boredom has been characterized as a cognitive signal that arises if a situation at hand is no longer promising as compared to possible alternatives^33,44^, promoting a behavioral switch to alternative actions. For the Boredom Choice Task, we therefore hypothesized that high boredom should lead to a stronger avoidance of the more monotonous alternative, as also suggested by previous work^30^. Importantly, as the task procedure is very repetitive per se, we did not suspect a noteworthy reduction of boredom, even when choosing the less monotonous option. This assumption was experimentally corroborated by the invariable induction of boredom in all participants after completing the task, independent of their choice. During the task, highly bored individuals seemed to strongly avoid the alternative with more sensory monotony, which however did not lead to a sufficient relief from boredom, as supported by the observed positive correlation of boredom bias and MSBS scores.

The link to boredom was further validated by showing that task experience matched participants’ imagined concepts of boredom in respect to affect and arousal, while being distinct from curiosity. Although this finding has to be treated with cautiousness, since self-reports of emotional components like e.g. arousal do often deviate from physiological measures of emotion, it points out the discreteness of boredom and curiosity as independent mental states^45,46^.

Despite the linkage to self-reported boredom experience, our approach projects the complex emotional state of boredom on a relatively simple behavior. Previous literature suggests that the human experience of boredom is multifaceted^24,35^ and, likely, our task does not capture all the facets of boredom by neglecting individual characteristics that do not map well to behavior. In our exploratory correlation analysis we mainly observed noteworthy interactions between behavior and the MSBS subscales of low-arousal and inattention. This suggests that, in line with the aforementioned intentions while designing the BCT, boredom leads to a behavioral response that tries to counteract the underchallenging monotonous sensory stimulation as cause of the low attention and arousal state. The separate study of all subdimensions of boredom and their link to different experiential and behavioral outcomes is worthy on its own. Still, while our task does correlate with several dimensions of boredom, its unidimensional measurement does not allow for the distinction between different dimensions. However, despite the issues with generalizing from the unidimensionality of our task, it does pave the road for a systematic and potentially translational investigation of the neural mechanisms underlying boredom.

### Neuroscientific outlook

A first line of potential future research is on the overall neural activity implicated in boredom, compared to activity observed during neutrality or engagement. Recent animal studies have shown that stimuli with greater salience increase cortical recruitment^47^. Building on these findings, future studies could examine whether an analogous phenomenon is demonstrated under boredom conditions. Explicitly, it could be tested whether different levels of experienced boredom lead to differential cortical activation. Our task provides a straightforward measurement tool for boredom-related behavior. A second line of potential research is on the specific brain mechanism implicated in boredom and subsequent behavior. In a recent imaging study in humans, activity in the anterior insula cortex was positively correlated with the default mode network (DMN) during an engaging condition, but anti-correlated with DMN during boring conditions that were either passive (watching a video) or active (detection of rare visual events). This differential boredom-dependent insular activity was suggested by the authors to represent a failure to engage executive control in a monotonous environment^48^. Our task provides a more controllable environment and a sensitive measurement of boredom-related decision making which can be utilized in future studies to explore the neural underpinning of boredom, and in particular the role of the insular cortex.

Our laboratory experimental cohorts were recruited from a homogeneous group of students that were largely young and healthy. It is thus not surprising that we did not detect any correlation between boredom bias and self-reports of mental health conditions. Nevertheless, future studies could use the Boredom Choice Task to study the interaction between boredom, deficient coping strategies with boredom and mental disorders such as ADHD and depression, that have well-established links to boredom^7–11^. In conclusion, the simplicity and non-verbal nature of the Boredom Choice Task represents a standardized framework for the study of boredom that can be used in healthy and clinical populations and, uniquely, in non-human species^34,49^. Compared to studies in humans, the translation to model organisms could enable investigations of the neural basis of boredom-related behavior, offering a wider spectrum of neural manipulations and measurements of brain activity.

### Model-based analysis of behavioral driving factors in the Boredom Choice Task

To understand the role of different factors determining the choices of individual subjects in the BCT, we developed a logistic regression model entailing two variables: Firstly, *entropy sensitivity*, reflecting the boredom-related avoidance of experienced monotony and secondly, a variable describing an *idiosyncratic choice bias* that also has commonly been described in two-alternative forced-choice tasks^36,41^.

The accuracy of the full regression model depended on the difference in entropy between both alternatives, with a maximal prediction accuracy of 77% (mean) for the individual choices made by a subject in the task condition of high entropy difference between alternatives. This degree of prediction accuracy indicates, that factors not captured by the model still have a substantial contribution to the decision behavior. For instance, a recent study found that decision-making while being bored shows more noise, in respect to a higher amount of switching between task alternatives, which is regarded as a motorical coping strategy with the monotony at hand^43^. Nevertheless, in the applied regression of our study *sensitivity to entropy* was identified as the most influential variable on model performance, indicating that sensory monotony, expressed as empirical entropy, constitutes the central property of the task that drives the boredom-related bias.

In comparison with various qualitative methods, which have been used to induce boredom^27,50^, the simplistic design of the BCT offered the opportunity to objectively measure the current situational monotony and to quantify it as *empirical entropy.* By observing that the difference in empirical entropy between the two alternatives serves as a major predictor for boredom-related choices in the task, we identify the degree of information content that is conveyed by the sensory stimulation as a direct driver for boredom-related behavior. Albeit this finding may appear intuitive, the development of a standardized behavioral paradigm both eliciting boredom and providing a parametric readout was essential to pinpoint the lack of information in the sensory input to the brain as a boredom factor.

### Boredom as a motivator to avoid low-information input states

Boredom has been characterized as a state of negative affect. Interestingly, this implies that boredom-inducing situations, such as environments offering only low information input to the brain, are avoided when possible. In these situations, boredom is therefore believed to act as a beneficial driver preventing individuals to get stuck and to seek novel information instead^33,51–53^. This assumption is corroborated by the results of the present study and furthermore shows congruence to other theories that identified boredom as an indicator of rising opportunity costs^54^ and emphasized it being a central mediator of exploration–exploitation tradeoffs in respect to predictive coding^52^. Interestingly, novelty-seeking is also associated with curiosity. However, curiosity is often directed towards a specific object or context^55^ and most importantly, it is associated with a positive affect^37^. Together, boredom and curiosity may work as complementary mechanisms guiding human behavior towards a stimulating environment, whereas boredom serves as a non-directed push-factor leading to the avoidance and overcoming of situations with low information level and curiosity serves as a directed pull-factor triggering the approach to high-information sources. In this scheme, the importance of information as an essential input to the brain is highlighted. Given the deleterious effects of long-term sensory deprivation and monotony on mental health^56–58^, boredom appears as a fundamental safeguard mechanism for the brain.

## Conclusion

In summary, the current study provides empirical evidence for a quantifiable boredom-related choice bias in a manipulable task environment together with a theoretical framework to interpret this setting and behavior. The major advantages of this approach are its simple and quantitative operationalization, its objectivity and the potential of translation to clinical and animal studies in basic research. In the present study, we leveraged on the Boredom Choice Task to identify the lack of information content of sensory stimulation being a key driver of boredom-related behavior.

## Materials and methods

The study was approved by the local ethics committee [Ethikkommission der Landesärztekammer Rheinland-Pfalz, processing number: 837.066.17 (10900)] and was conducted in accordance with the Declaration of Helsinki. Written informed consent was obtained from the participants of the study.

### Task details

The *Boredom Choice Task* (BCT) that was utilized in all four experiments operationalized aversion as the central property of boredom experience^1,33–35^. Concretely, the task measured behavioral avoidance of one of two alternatives that both were associated with sensory stimulation of different degrees of monotony. We implemented the BCT in a custom *php* software accessible through a standard internet browser. The two alternatives of the BCT consisted of mirrors-image buttons which were located on opposite corners of a computer screen. Participants were placed in front of the screen and received an instruction, asking them to choose between alternatives with the computer mouse. The key term *boredom* was not mentioned in the introduction. After clicking on either of them, both buttons disappeared for 1 s and a sensory stimulus was presented (either *visual stimuli*: images of everyday objects, or *auditory stimuli*: sound recordings of spoken German words). For the new trial, the buttons reappeared in contrary corners, so that subjects on each trial had to move the computer mouse to decide for one button anew, in order to control for extensive switching behavior that would likely interfere with boredom experience^43^. After completing the task, participants received a pre-determined monetary reward independent from task performance.

For the visual task a stimulus pool was used, containing 450 images of everyday objects from the Bank of Standardized Stimuli^59,60^, whereas the auditory stimulus pool comprised 300 neutral spoken German words downloaded via the website https://soundoftext.com (for representative examples of the stimuli see Fig. 1A,B).

Task cycles, comparing two alternatives with particular degrees of monotony, built the core of each of the experiments, conducted in this study. The Boredom Choice Task design derived from the hypothesis that the extent of the avoidance of the more monotonous alternative reflected state boredom.

Participants furthermore completed a list of standard psychometric assessments that quantified state boredom (*VAS-B*: a visual analog scale for state boredom in a 100 step grading (see Additional Information for a screenshot), *MSBS*: Multidimensional State Boredom Scale^35^ with an annotation that all questions referred to the feeling during the BCT (the internal consistency of this slightly modified MSBS version was comparable to previous studies^35,61^: Cronbach’s α of n = 250 participants: sum score: 0.94, disengagement: 0.87, low arousal 0.79, high arousal: 0.81, inattention: 0.76, time perception: 0.91), trait boredom (*BPS*: Boredom Proneness Scale^62^), personality structure (*BFI-10*: Big Five Inventory^63^, *BRS*: Brief Resilience Scale^64,65^) as well as symptoms of mental disorders (*GHQ-28*: General Health Questionnaire^66^, *CAARS:S-L*: Conner’s Adult ADHD Rating Scale^67^, *BDI-II*: Beck’s Depression Inventory^68^, I-8: Impulsivity Questionnaire^69,70^, *STAI-Y*: State Trait Anxiety Inventory^71^) and general information of sociodemographic background and patient history (*GI*: general information). In addition, visual analog ratings of affect and arousal^72^ (*VAS-AA*) were applied in which participants rated an imagined situation of *boredom* and *curiosity* as well as their *experience during the BCT* (see Additional Information for a screenshot).

### Experimental procedure

#### Experiment Ia

49 healthy undergraduate students from the university of Mainz were recruited via an online recruiting system^73^. Each subject received an expense allowance of 25 € for participation. Exclusion criteria were: active mental disorders, hearing loss, strongly impaired vision and insufficient language knowledge. All recruited individuals fulfilled these requirements and represented a healthy sample with young adult age (for sociodemographic information see Supplementary Table 1). The experiment was conducted in the Mainz Behavioral and Experimental Laboratory, where subjects were welcomed, instructed to the experiment and hereupon completed different self-report scales (BPS, BFI-10, BRS, GHQ-28, CAARS:S-L, BDI-II, I-8, STAI-Y, GI) before executing a total of six BCT cycles of 300 trials length each (*condition*, size of stimulus libraries [size_alternative1_:size_alternative2_]: visual: monotonous vs. monotonous (*Mon–Mon*) 1:1, variable vs. variable (*Var–Var*) 225:225, monotonous vs. variable (*Mon–Var*) 1:449; auditory: *Mon–Mon* 1:1, *Var–Var*, 150:150, *Mon–Var* 1:299; see Fig. 1B). To avoid potential biases due to prior task experience in the condition with maximally different alternatives, the two *Mon–Var* BCT cycles implementing maximal difference between alternatives, were conducted prior to the four control cycles (*Mon–Mon* and *Var–Var*, each with visual and auditory stimuli). The order of BCT cycles within these two sets was random. After completing all six BCT cycles, participants rated their state boredom during the task on the MSBS. The experimental procedure took approximately 2.5 h in total.

#### Experiment Ib

53 different, BCT-naïve participants were recruited through the same procedure as in Experiment Ia (for sociodemographic information see Supplementary Table 1). The experimental procedure was also equivalent except an addition of a visual analog scale to rate current state boredom before and after the BCT cycles (*VAS-B*) (see Fig. 1B,C). Furthermore visual analog ratings of affect and arousal^72^ for imagined states of *boredom* and *curiosity* were conducted prior to the task (*VAS-AA*). After the task, an equivalent rating of affect and arousal was conducted that asked participants to rate their *experience during the BCT*.

#### Experiment Ic

Thirdly, 40 participants were recruited via the online platform *Amazon Mechanical Turk *(https://mturk.com) to complete the BCT under online experimental conditions. These participants were uncontrolled in regards to their sociodemographic background and mental health. After completion of the experiment, participants received a monetary amount of 5 $ (USD). In order to reduce the length of the experiment, online subjects only completed three BCT cycles with visual stimuli (*Mon–Mon*, *Var–Var*, *Mon–Var*), however the task settings (e.g. duration and sensory stimuli) were equivalent to Experiment Ia and b. From the list of questionnaires only the BPS was rated prior to the BCT cycles and the MSBS was assessed after the task. The experimental procedure took approximately 1 h in total.

#### Experiment II

148 participants were recruited equivalently to the laboratory Experiments Ia and b. All recruited subjects fulfilled the inclusion criteria and qualitatively matched the sociodemographic characteristics of the previous laboratory cohorts (see Supplementary Table 1). Participants were presented with the same list of questionnaires and visual analog scales as in Experiment Ib, expanded to include a *working memory test* (digit span backwards task^74^) prior to the BCT. The behavioral probe comprised 13 BCT cycles, each of 100 trials length. The BCT cycles all included *visual stimuli* only, but differed in the repetitiveness of sensory stimulation associated to each button (size of stimulus libraries [size_alternative1_:size_alternative2_]: 1:1, 2:1, 4:1, 8:1, 16:1, 32:1, 64:1, 64:2, 64:4, 64:8, 64:16, 64:32, 64:64; see Fig. 4A). The order of all these 13 BCT cycles was random. The experimental procedure took approximately 2 h in total.

### Statistical analysis

All analyses were conducted using the MATLAB® statistics and machine learning toolbox (The Mathworks Inc., Natick, Massachusetts, USA, version R2016b).

#### Questionnaires

The self-report data was analyzed by computing the sum score for each questionnaire. Subjects that accidentally skipped single items of a questionnaire were excluded from the respective analysis.

#### Analysis of the choice bias

As a simple readout for behavioral boredom in the BCT, we computed the amount of choices for the less monotonous alternative relative to all trials of one BCT cycle (*raw boredom bias* ${\uppi }$). For the cycles with equivalent monotony for both alternatives (*Mon–Mon, Var–Var*) the right-located alternative was chosen as reference:

$$raw\, bias = b = \frac{{n_{choices\, variable/right} }}{{n_{all\, choices} }}$$

To find a refined measure of individual monotony avoidance, the *raw boredom bias* was averaged over the *Mon–Var* cycles in visual and auditory modality ($\overline{{b_{MonVar} }} )$ and consecutively standardized according to each individual’s average *idiosyncratic choice bias* in the four control BCT cycles ($\overline{{b_{Control} }}$ referring to an *idiosyncratic bias* for the right alternative). Therefore, both average bias scores were combined by subtracting the idiosyncratic bias from the boredom bias of highly unequal alternatives:

$$B = \overline{{b_{MonVar} }} - \overline{{b_{Control} }}$$

This operation yielded an *adjusted boredom bias* $B$ with values ranging theoretically from − 1 (completely preferring the monotonous/left alternative in the *Mon–Var* cycles in contrast to an opposite *idiosyncratic bias*) over 0 (no bias for either side) to 1 (completely avoiding the monotonous/left alternative in the *Mon–Var* cycles in contrast to an opposite *idiosyncratic bias*).

By way of example, a subject could undergo both *Mon–Var* BCT cycles with the variable alternative being positioned on the right side and show an average *raw boredom bias* of $\overline{{b_{MonVar} }} = 0.9$. In the four control BCT cycles this subject could show an average *idiosyncratic bias* for the right alternative of $\overline{{b_{Control} }} = 0.6$. This example would then correspond to an *adjusted boredom bias* of $B = 0.9 - 0.6 = 0.3$.

#### Computation of empirical entropy

*Empirical entropy* was computed for each alternative in the BCT as a measure of current information over all the stimuli that were experienced by a participant up to a given trial in a BCT cycle:

$$Entropy_{j, t} = H_{j, t} = - \mathop \sum \limits_{i = 1}^{{n_{j, t} }} f_{i, j, t} *\log \left( {f_{i,j,t} } \right)$$

where (i) $n_{j, t}$ represents the number of different unique stimuli that were obtained from sampling alternative $j$ up to trial $t$*,* and (ii) $f_{i, j, t}$ is the relative frequency of presenting stimulus $i$ at alternative $j$ on trial $t$. This method quantifies entropy for each alternative at a given trial as a fraction of the total entropy provided by both alternatives (see Supplementary Fig. 5). The obtained numerical value reflects the current state of variety within the distribution of experienced stimuli from a specific alternative. If both alternatives present only one stimulus, the entropy of an alternative becomes lower, as it is chosen more frequently. On the other side, if both alternatives present multiple stimuli, the entropy value of an alternative increases as it is chosen more frequently. If one alternative is never chosen, its entropy is set to zero.

#### Linear regression model for choice behavior during the task

To describe individual choice probability in each trial of the BCT based on experienced empirical entropy and idiosyncratic bias, we considered the following logistic regression model:

$$P_{t} \left( {right} \right) = \frac{1}{{1 + e^{{ - {\uptheta }}} }}$$

where $P_{t} \left( {right} \right)$ is the probability of choosing the right positioned alternative based on an individual’s experience up to trial $t$ and.

$${\uptheta } = { }w_{0} {\Delta }H_{t} + w_{1}.$$

In this model $w_{i} , i \in \left\{ {0 \ldots 1} \right\}$ are the fitted parameter weights for each participant. A maximum likelihood estimation was conducted to find the best fitting parameter weights $w_{0}$ and $w_{1}$ for the 1287 data pairs of entropy difference and consecutive choice that each participant in Experiment II yielded; ${\Delta }H_{t}$ is the *difference in entropy* between the two alternatives in the current trial, where the entropy of the left alternative is subtracted from the right alternative’s entropy. This value together with the weight $w_{0}$ reflects a *sensitivity to entropy* in the model; $$right = \left\{ {\begin{array}{*{20}l} {1\, if\, right} \\ {0\, if\, left} \\ \end{array} } \right.$$ is the binary indicator of whether current choice is in favor of the right positioned alternative. Together with the parameter weight $w_{1}$ it reflects the parameter *idiosyncratic bias*.

To investigate the influence of the different parameters, the full model was compared with partial models that only included one parameter. We applied a model with ${\uptheta }$ depending on entropy sensitivity only (${\uptheta }_{{\text{only entropy sensitivity}}} = { }w_{0} {\Delta }H_{t}$) and with $\uptheta$ depending on the idiosyncratic bias only (${\uptheta }_{{\text{only idiosyncratic bias}}} = { }w_{1}$). Again, a maximum likelihood estimation was conducted to find the best fitting parameter weights.

#### Correlation analysis

In order to investigate the link between task behavior and self-reported boredom experience as well as psychometric properties, we conducted a stepwise correlation analysis. In a first step, we exploratively correlated the adjusted boredom bias to all psychometric and boredom specific assessments without significance testing of particular hypotheses (Experiment Ia). Based on the findings of our exploratory analysis, in a second step, we replicated the experiment with a cohort of independent participants (Experiment Ib) and specifically tested only the single correlation of boredom bias to MSBS sum score that previously showed a relevant effect size for significance. The size of this second cohort was determined by a sample size computation assuming the previously observed correlation strength and a power level of 0.8.

## Supplementary Information

Supplementary Information.

## Data Availability

The data of this study is available from the corresponding authors upon reasonable request.
