# Supplementary material for: Experienced entropy drives choice behavior in a boring decision-making task
Source: Sci Rep. 2022 Feb 24;12:3162. doi: 10.1038/s41598-022-06861-w (PMC8873446; doi:10.1038/s41598-022-06861-w)
Supplement: Supplementary file 1 — Supplementary Information. [file 41598_2022_6861_MOESM1_ESM.pdf]

Experienced entropy drives choice behavior in a boring decision-making task

Johannes P.-H. Seiler[1,2,*], Ohad Dan[3,*], Oliver Tüscher[2,4,**], Yonatan Loewenstein[5,**], Simon Rumpel[1,**]

[1] Institute of Physiology, University Medical Center of the Johannes Gutenberg University Mainz, Hanns-Dieter-Hüsch-Weg 19, 55131 Mainz, Germany

[2] Department of Psychiatry and Psychotherapy, University Medical Center of the Johannes Gutenberg University Mainz, Untere Zahlbacher Straße 8, 55131 Mainz, Germany

[3] Department of Comparative Medicine, Yale School of Medicine, New Haven CT, 06520, USA

[4] Leibniz Institute for Resilience Research, Wallstraße 7, 55122 Mainz, Germany

[5] The Alexander Silberman Institute of Life Sciences, Department of Cognitive Sciences, The Federmann Center for the Study of Rationality, The Hebrew University of Jerusalem, Jerusalem, Israel 9190401

[*] First authors that contributed equally

[**] Senior authors that contributed equally

Supplementary Information

Supplementary Figures

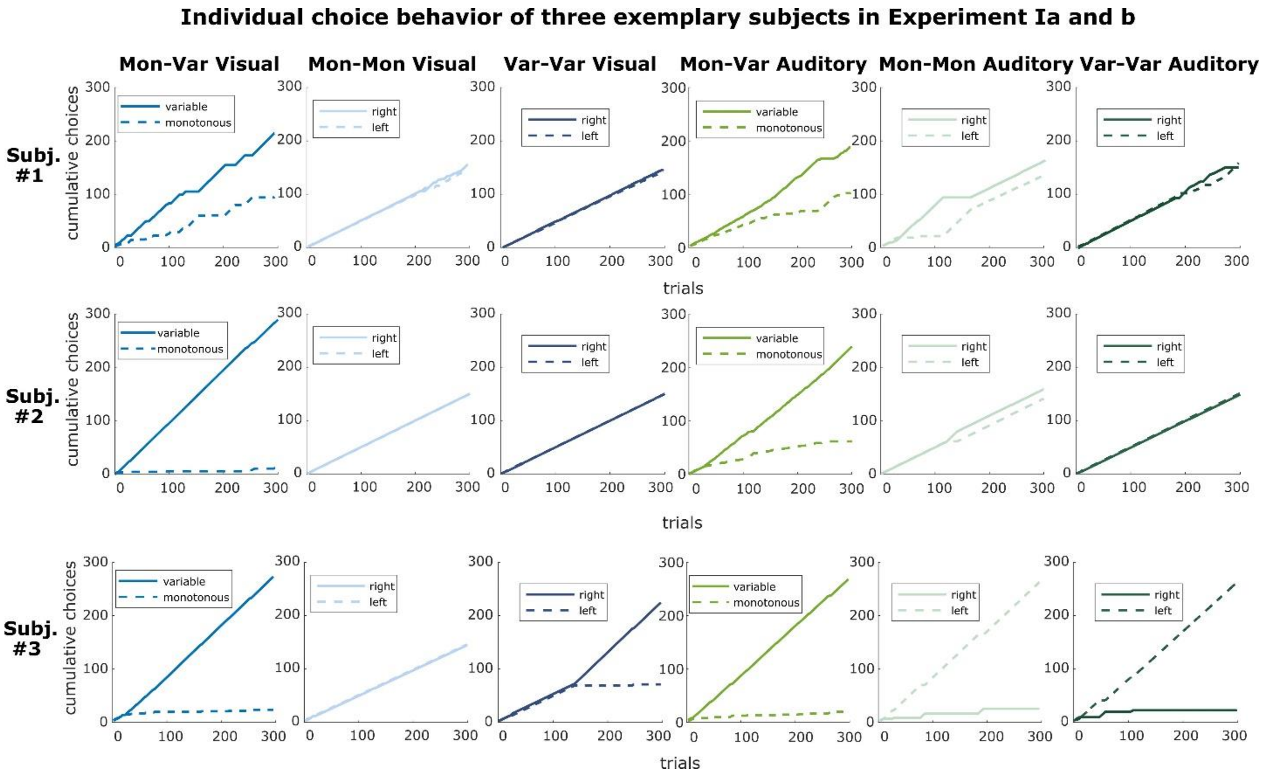

Supplementary Figure 1 – Choice behavior of three exemplary subjects from Experiment Ia and b in all BCT conditions: The cumulative amount of choices for either alternative is plotted for each trial and the color indicates the respective task condition. Different subjects show a different magnitude of their *boredom bias* (e.g. compare Subj. 1 and Subj 2 in *Mon-Var* Auditory) and also vary in choice behavior from visual to the auditory task cycles (e.g. compare Subj. 1 *Mon-Var* Auditory and *Mon-Var* Visual). In the control conditions, subjects either switch regularly between alternatives (e.g. Subj. 3 in *Mon-Mon* Visual), or show an idiosyncratic bias for one of the equivalent alternatives (e.g. Subj. 3 in *Mon-Mon* Auditory, in this case with an idiosyncratic bias for the left alternative).

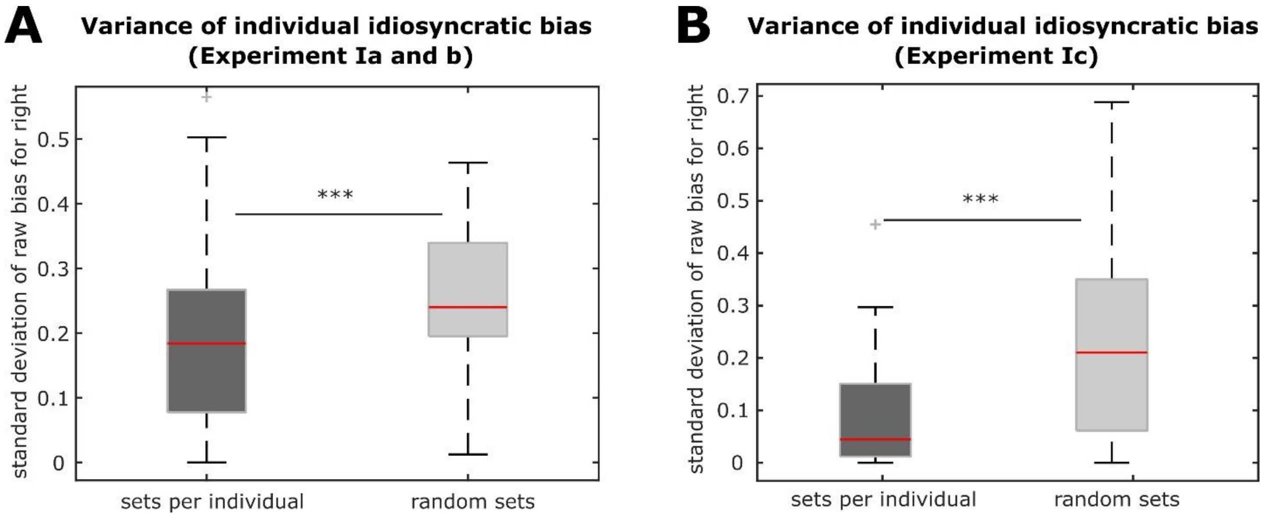

Supplementary Figure 2 – Subjects show an idiosyncratic bias for one alternative in the symmetric BCT cycles that reduces variance within the behavior in different BCT cycles: (**A**) Boxplots of the standard deviation of each individual's *raw boredom biases* for the alternative located at the right side of the screen in the four control cycles from the laboratory Experiments Ia and b (n = 102 participants, each with *monotonous vs. monotonous* and *variable vs variable* task cycles in visual and auditory modality). Individuals show less variance in their bias compared to randomly sampling four bias scores across individuals. This hints towards a systematic *idiosyncratic bias* on the level of single subjects. Both conditions show a significant statistical difference (Wilcoxon ranked sum test with ***: $p < 0.001$). (**B**) Equivalent plot for the *raw boredom bias* in the online Experiment Ic (n = 40 participants, each with one *monotonous vs. monotonous* and *variable vs variable* task cycle in visual modality), where the finding of an individual *idiosyncratic bias* is confirmed.

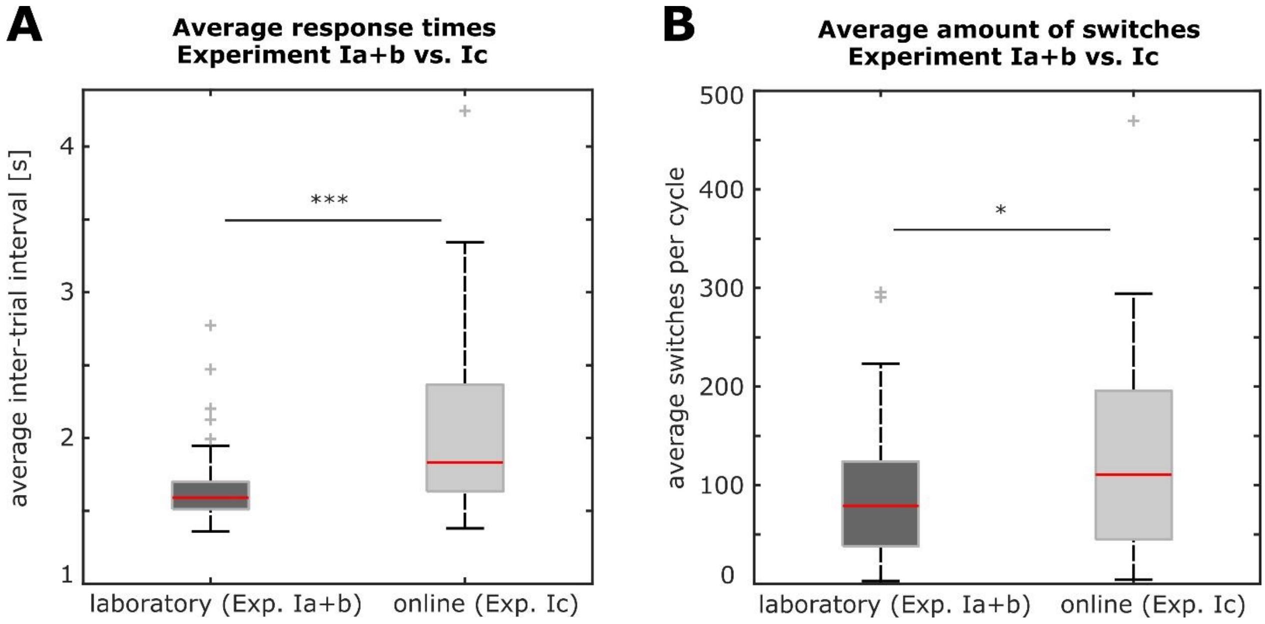

Supplementary Figure 3 – Comparison of laboratory and online experiments: Comparing the extent of the boredom bias in the BCT under the controlled conditions of a laboratory (Experiments Ia and b*)* against less controlled online conditions (Experiment Ic), we found that the bias was less strongly pronounced in the online environment (Figure 2C). We hypothesized that this difference may be produced by less task adherence, potentially due to a more distracting environment when conducting the experiment online. Therefore, for each individual we computed the average *inter-trial latency* as well as the average *amount of switches* in one BCT cycle, and compared these behavioral metrics between the laboratory experiments and the online experiment. We operationalize these behavioral outcomes as indirect representatives for adherence and attention to the BCT. (**A**) Boxplots of average response latencies from the laboratory studies (Experiment Ia and b) and online study (Experiment Ic). Online subjects show longer response latencies compared to the subjects from the laboratory study (median ± SD for individuals' average inter-trial latency: laboratory Experiments Ia+b: 1.59s ± 0.21s, n = 102 participants; online Experiment Ic: 1.83s ± 0.62s, n = 40 participants; Wilcoxon ranked sum test with ***: $p < 0.001$). The stimulus presentation time was set to 1 s, which explains the minimal values. (**B**) Boxplots of the average amount of switches per BCT cycle. Online subjects show significantly more switching between alternatives compared to the subjects from the laboratory study (median ± SD for individuals' average amount of switching: laboratory Experiments Ia+b: 79.08 ± 60.55, n = 102 participants; online Experiment Ic: 110.67 ± 96.62, n = 40 participants; Wilcoxon ranked sum test with *: $p = 0.049$). Both findings together indicate that subjects under online conditions are less adherent to the task (reflected by increased inter-trial intervals) and generally show a more alternating behavior (reflected by more frequent switching).

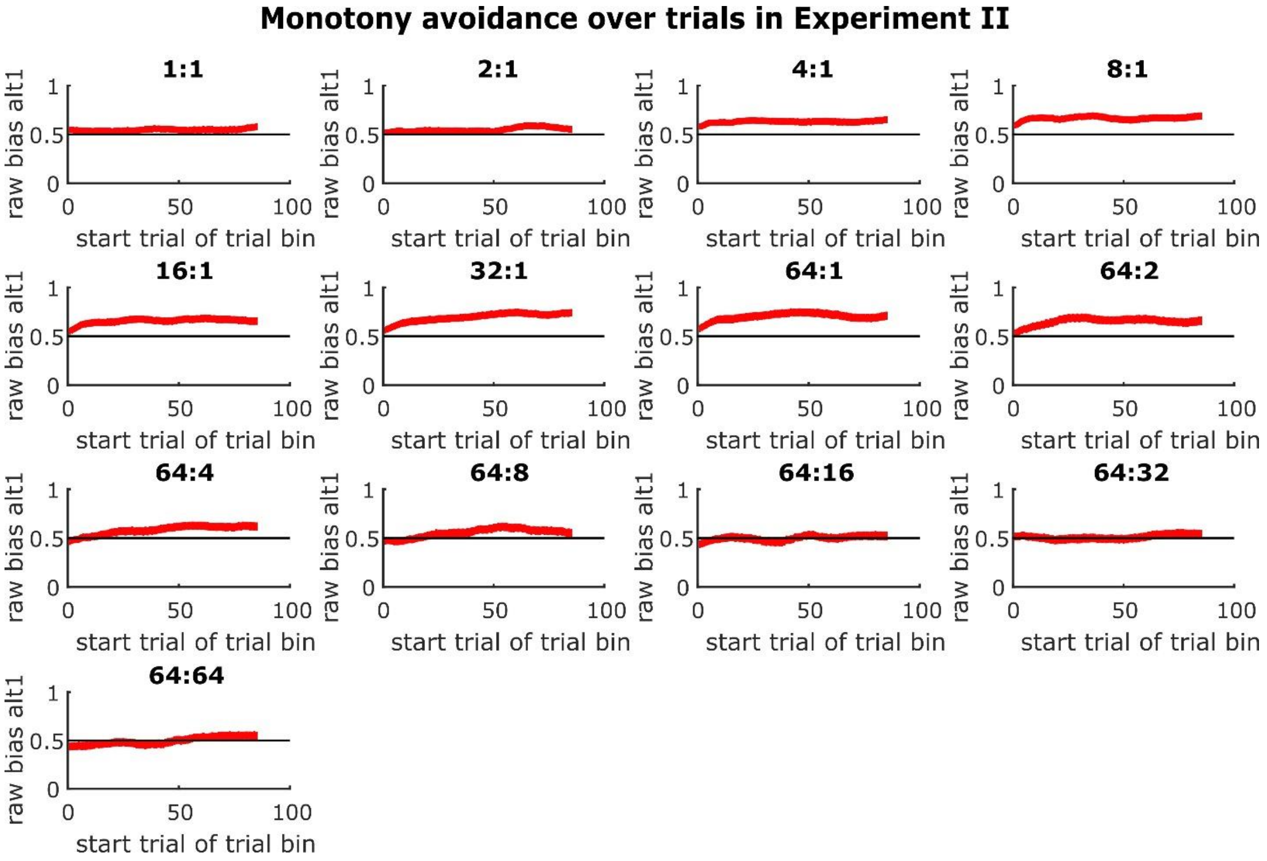

Supplementary Figure 4 – Raw boredom bias profiles and adaptations across experiments: In Experiment II each subject completed 13 cycles of the BCT with different library sizes of visual stimuli. Each panel presents the average *raw boredom bias* over the trials of each task cycle (n = 148 participants). The *raw boredom bias* of each individual is computed in a bin of 15 trials that is then shifted stepwise until the end of the task (first bin: trial 1-15, last bin: trial 86-100). The average adaptation curves of the boredom bias qualitatively match the adaptation curves from Experiment Ia-c (see Figure 2B) with an initial increase of the boredom bias followed by a stable plateau phase. The magnitude of the maximal boredom bias increases as the difference between the two stimulus libraries becomes larger. Despite the different length of the BCT cycles in Experiment II with only 100 trials, the similar adaptation curves in Experiment II support a general comparability with the previous Experiments Ia-c with 300 trials.

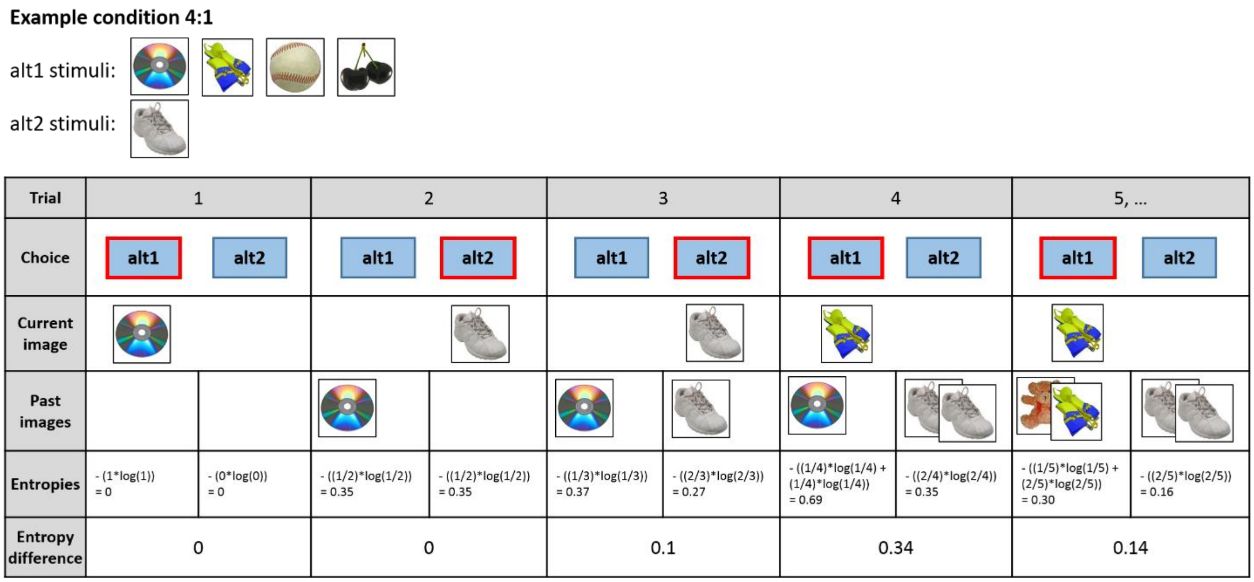

Supplementary Figure 5 – Schematic example of entropy computation: The table illustrates the computation of *empirical entropy* for the first 5 trials of an exemplary 4:1 stimuli BCT condition. The red frame marks the alternative which is chosen in each trial. In addition, the stimuli of the current trial and past trials are presented. Note that in this example not all possible stimuli from the libraries are sampled. Entropy for each alternative is computed on each trial as a fraction of the total entropy provided by the stimuli of both alternatives. If one alternative is never chosen, its entropy is set to zero. To compare the state of entropy between both alternatives we furthermore computed the *difference in entropy* for each trial.

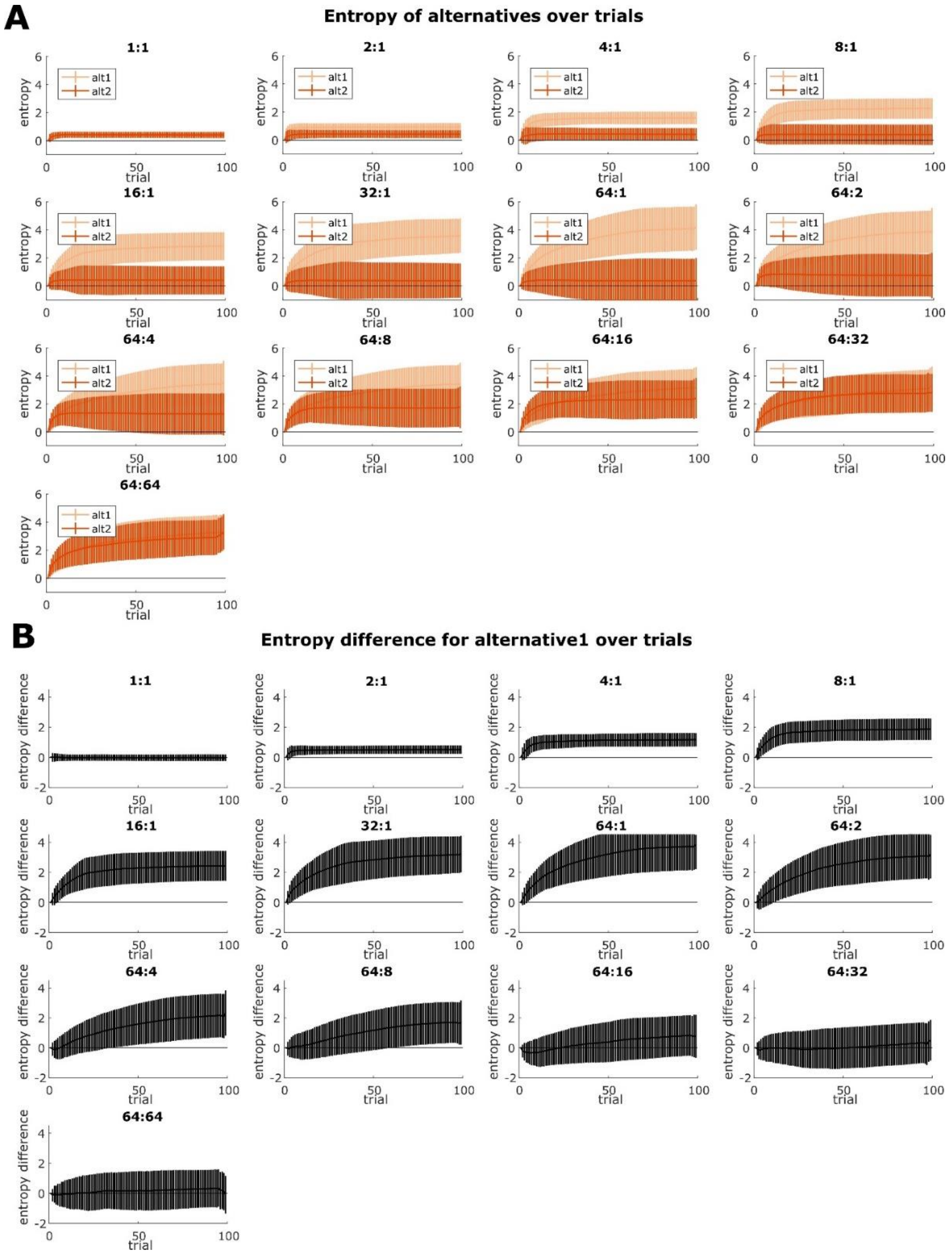

Supplementary Figure 6 – Empirical entropy over trials in the different BCT cycles of Experiment II: (**A**) Average *empirical entropy* for the two alternatives, computed over all stimuli that were experienced at the respective alternative up to the current trial (n = 148 participants). The vertical bars indicate the standard error of the mean. *Empirical entropy* of each alternative is computed for each trial and therefore evolves over the duration of the task cycles. (**B**) Average *entropy difference* between the two alternatives in each trial of the task cycles in Experiment II (n = 148 participants). The difference in empirical entropy depends on the difference between the stimulus libraries and is largest for the 64:1 condition. The vertical bars indicate the standard error of the mean.

Supplementary tables

	**Experiment Ia** (n = 49)	**Experiment Ib** (n = 53)	**Experiment II** (n = 148)
**Gender**			
Female	34 (69.4%)	32 (60.4%)	114 (78.0%)
Male	15 (30.6%)	21 (39.6%)	34 (23.0%)
**Age** (years)			
Mean	24.6	21.6	22.1
Standard deviation	4.7	2.8	3.0
**Body weight** (kg)			
Mean	68.1	67.5	68.3
Standard deviation	11.4	11.5	12.6
**Height** (cm)			
Mean	172.4	173.5	172.1
Standard deviation	9.3	9.1	9.5
**BMI** (kg/m^2)			
Mean	22.8	22.4	23.0
Standard deviation	2.6	2.8	3.5
**Ethnical background**			
Caucasian	44 (89.8%)	51 (96.2%)	141 (95.3%)
African	1 (2.0%)	1 (1.9%)	3 (2.0%)
Asian	5 (10.2%)	4 (7.5%)	4 (2.7%)
Other	1 (2.0%)	1 (1.9%)	2 (1.4%)
**Size of hometown** (inhabitants)			
< 10 000	16 (32.7%)	22 (41.5%)	71 (48.0%)
10 000 – 100 000	21 (42.9%)	18 (34.0%)	48 (32.4%)
> 100 000	12 (24.5%)	13 (24.5%)	29 (19.6%)
**Subject of studies**			
Economics	13 (26.5%)	13 (24.5%)	33 (22.3 %)
Business administration	2 (4.1%)	1 (1.9%)	1 (0.7%)
Law	7 (14.3%)	6 (11.3%)	9 (6.1%)
Teaching post	4 (8.2%)	5 (9.4%)	18 (12.2%)
Sociology	2 (4.1%)	1 (1.9%)	7 (4.7%)
Biology	4 (8.2%)	4 (7.5%)	6 (4.1%)
Medicine	1 (2.0%)	3 (3.8%)	8 (5.4%)
Journalism	1 (2.0%)	4 (7.5%)	5 (3.4%)
Politics	1 (2.0%)	2 (3.8%)	7 (4.7%)
Other	14 (28.6%)	15 (28.3%)	54 (36.5%)
**Mental disorder in family**			
Yes	13 (26.5%)	13 (24.5%)	49 (33.1%)

Supplementary Table 1 – Sociodemographic characteristics of participants from Experiments Ia+b and II: A pooled analysis of all participants from the three laboratory experiments (Experiment Ia+b and II) shows an average age of 23 years and a mean BMI of 22.7 kg/m2 corresponding to normal average weight. The majority of sampled individuals is female (72.0%), has a Caucasian background (94.8%) and stems from a small hometown with less than 10,000 inhabitants (43.6%). All participants are enrolled students from the Johannes-Gutenberg University Mainz, where the most frequent fields of study covered economics (23.6%), teaching post (10.8%) and law (8.8%). None of the participants suffered from active mental disorders, however a substantial fraction reported diagnosed mental disorders in the close family (30.0%). All in all, these results indicate a homogenous sample of young and healthy adults with a higher-than-average education.

	**BCT experience vs. Imagined Boredom**	**BCT experience vs. Imagined Curiosity**	**Imagined Boredom vs. Imagined Curiosity**
**Affect**	**R = 0.32**, p = 0.02	R = 0.06, p = 0.69	R = -0.16, p = 0.25
**Arousal**	**R = 0.48**, p < 0.001	R = 0.04, p = 0.78	R = -0.02, p = 0.91

Supplementary Table 2 – Correlations of affect and arousal ratings: Spearman correlations of the visual analog ratings of *imagined curiosity, imagined boredom* and the *BCT experience*. Each correlation is computed over n = 49 participants from Experiment Ib. Bold letters indicate statistically significant correlation coefficients with $p < 0.05$.

**Correlations of adjusted boredom bias and working memory capacity**													
**Stimulus libraries of task cycle**	**1:1**	**2:1**	**4:1**	**8:1**	**16:1**	**32:1**	**64:1**	**64:2**	**64:4**	**64:8**	**64:16**	**64:32**	**64:64**
**Spearman's R**	-0.07	0.05	0.10	-0.12	0.12	-0.03	-0.03	-0.08	-0.04	-0.13	-0.03	0.02	0.07
**p**	0.54	0.68	0.38	0.30	0.31	0.83	0.81	0.50	0.72	0.26	0.79	0.85	0.55

Supplementary Table 3 – Correlations of adjusted boredom bias and working memory capacity: Spearman correlations between the *adjusted boredom bias* and the *working memory task scores* (digit span backwards task) for the subjects of Experiment II that completed the working memory task (n = 72 participants). Due to the plateau level of the boredom bias for alternatives with stimulus libraries larger than 8 stimuli (see Figure 4B), we hypothesized that *working memory capacity*, which is typically reported to cover around 7 chunks of information, could moderate the amplitude of the boredom bias in the BCT. Therefore, we tested the correlation of *working memory task scores* and the *adjusted boredom bias* of monotony avoidance in all 13 task cycles of Experiment II. We did not observe a significant relationship for any of the task cycles, indicating that differences in working memory capacity are not able to explain the differences in the magnitudes of adjusted boredom bias. Note, for the symmetrical conditions 1:1 and 64:64 by construction no correlation would have been expected.

German versions of self-report boredom assessments

Boredom Proneness Scale (BPS)

BPS

Die folgenden Aussagen können mehr oder weniger auf Sie zutreffen. Bitte geben Sie bei jeder Aussage an, inwieweit diese auf Sie persönlich zutrifft.

	trifft überhaupt nicht zu 1	2	3	4	5	6	trifft vollkommen zu 7
1. Es fällt mir leicht, mich auf meine Aktivitäten zu konzentrieren.	○	○	○	○	○	○	○
2. Während ich arbeite, mache ich mir oft Sorgen um andere Dinge.	○	○	○	○	○	○	○
3. Die Zeit scheint immer nur langsam zu verstreichen.	○	○	○	○	○	○	○
4. Ich weiß oft nichts mit mir anzufangen.	○	○	○	○	○	○	○
5. Ich gerate oft in Situationen, in denen ich mich mit sinnlosen Dingen beschäftigen muss.	○	○	○	○	○	○	○
6. Es langweilt mich enorm, Heimvideos oder Urlaubsfotos anderer anzuschauen.	○	○	○	○	○	○	○
7. Ich beschäftige mich gedanklich immerzu mit Projekten bzw. Dingen, die erledigt werden müssen.	○	○	○	○	○	○	○
8. Es fällt mir leicht, mich zu beschäftigen.	○	○	○	○	○	○	○
9. Viele Dinge, die ich tun muss, sind repetitiv und monoton.	○	○	○	○	○	○	○
10. Es braucht mehr Stimulation, um mich in Gang zu bringen, als dies bei den meisten Menschen der Fall ist.	○	○	○	○	○	○	○
11. Die meisten Dinge, die ich tue, machen mir großen Spaß.	○	○	○	○	○	○	○
12. Meine Arbeit begeistert mich nur selten.	○	○	○	○	○	○	○
13. Ich kann in der Regel in jeder Situation etwas zu tun oder zu sehen finden, das mich interessiert.	○	○	○	○	○	○	○
14. Die meiste Zeit sitze ich nur herum und tue nichts.	○	○	○	○	○	○	○
15. Ich kann gut geduldig warten.	○	○	○	○	○	○	○
16. Ich habe oft nichts zu tun und zu viel Zeit.	○	○	○	○	○	○	○
17. In Situationen, in denen ich warten muss, z. B. in einer Warteschlange, werde ich sehr unruhig.	○	○	○	○	○	○	○
18. Ich wache oft mit einer neuen Idee auf.	○	○	○	○	○	○	○
19. Es wäre sehr schwierig für mich, eine Arbeitsstelle zu finden, die interessant genug ist.	○	○	○	○	○	○	○
20. Ich wünsche mir mehr Herausforderungen im Leben.	○	○	○	○	○	○	○
21. Ich bin der Meinung, dass ich die meiste Zeit weit unter meinen Fähigkeiten arbeite.	○	○	○	○	○	○	○
22. Viele Leute würden sagen, dass ich kreativ und einfallsreich bin.	○	○	○	○	○	○	○
23. Ich habe so viele Interessen, dass ich keine Zeit habe, allen nachzugehen.	○	○	○	○	○	○	○
24. In meinem Freundeskreis bin ich die Person, die sich am längsten mit einer Sache beschäftigt.	○	○	○	○	○	○	○

	1	2	3	4	5	6	7
25. Wenn ich nichts Aufregendes oder sogar Gefährliches mache, fühle ich mich halbtot und langweilig.	○	○	○	○	○	○	○
26. Es braucht viel Abwechslung, um mich wirklich glücklich zu machen.	○	○	○	○	○	○	○
27. Es scheint, dass im Fernsehen oder im Kino immer die gleichen Sachen laufen; das nervt langsam.	○	○	○	○	○	○	○
28. Als ich jung war, fand ich mich oft in monotonen und langweiligen Situationen wieder.	○	○	○	○	○	○	○

Translation of R. Farmer & N. D. Sundberg, 1986

Multidimensional State Boredom Scale (MSBS)

MSBS

Die folgenden Aussagen beziehen sich auf ihr **persönliches Empfinden während des eben bearbeiteten Computertests**. Bitte geben Sie bei jeder Aussage an, inwieweit diese auf Sie **während des Tests** zutraf.

	trifft überhaupt nicht zu 1	2	3	4	5	6	trifft vollkommen zu 7
1. Die Zeit vergeht langsamer als gewöhnlich.	○	○	○	○	○	○	○
2. Ich stecke in einer Situation fest, die ich belanglos finde.	○	○	○	○	○	○	○
3. Ich lasse mich leicht ablenken.	○	○	○	○	○	○	○
4. Ich bin einsam.	○	○	○	○	○	○	○
5. Zurzeit regt mich scheinbar alles auf.	○	○	○	○	○	○	○
6. Ich wünschte, die Zeit würde schneller vergehen.	○	○	○	○	○	○	○
7. Alles erscheint mir monoton und alltäglich.	○	○	○	○	○	○	○
8. Ich fühle mich niedergeschlagen.	○	○	○	○	○	○	○
9. Ich scheine gezwungen zu sein, Dinge zu tun, die für mich keinen Wert haben.	○	○	○	○	○	○	○
10. Ich fühle mich gelangweilt.	○	○	○	○	○	○	○
11. Die Zeit zieht sich hin.	○	○	○	○	○	○	○
12. Ich bin launischer als sonst.	○	○	○	○	○	○	○
13. Ich bin unentschlossen oder unsicher, was ich als nächstes tun soll.	○	○	○	○	○	○	○
14. Ich fühle mich aufgewühlt.	○	○	○	○	○	○	○
15. Ich fühle mich leer.	○	○	○	○	○	○	○
16. Es fällt mir schwer, mich zu konzentrieren.	○	○	○	○	○	○	○
17. Ich möchte etwas machen, was Spaß macht, aber nichts spricht mich an.	○	○	○	○	○	○	○
18. Die Zeit verstreicht nur langsam.	○	○	○	○	○	○	○
19. Ich wünschte, ich würde etwas Aufregenderes machen.	○	○	○	○	○	○	○
20. Meine Aufmerksamkeitsspanne ist kürzer als gewöhnlich.	○	○	○	○	○	○	○
21. Ich bin gerade ungeduldig.	○	○	○	○	○	○	○
22. Ich verschwende Zeit, die ich besser auf etwas anderes verwenden sollte.	○	○	○	○	○	○	○
23. Meine Gedanken wandern.	○	○	○	○	○	○	○
24. Ich möchte, dass etwas passiert, aber ich bin mir nicht sicher, was.	○	○	○	○	○	○	○

	1	2	3	4	5	6	7
25. Ich fühle mich vom Rest der Welt abgeschnitten.	○	○	○	○	○	○	○
26. Im Moment scheint die Zeit nur langsam zu verstreichen.	○	○	○	○	○	○	○
27. Ich ärgere mich über die Leute in meinem Umfeld.	○	○	○	○	○	○	○
28. Mir kommt es vor, als säße ich herum und wartete darauf, dass etwas passiert.	○	○	○	○	○	○	○
29. Es scheint, als gäbe es niemanden, mit dem ich reden könnte.	○	○	○	○	○	○	○

Translation of S. A. Fahlman, K. B. Mercer-Lynn, D. B. Flora, J. D. Eastwood, 2013

Visual analog assessments of the study

Visual analog scale for state boredom in German language (VAS-B)

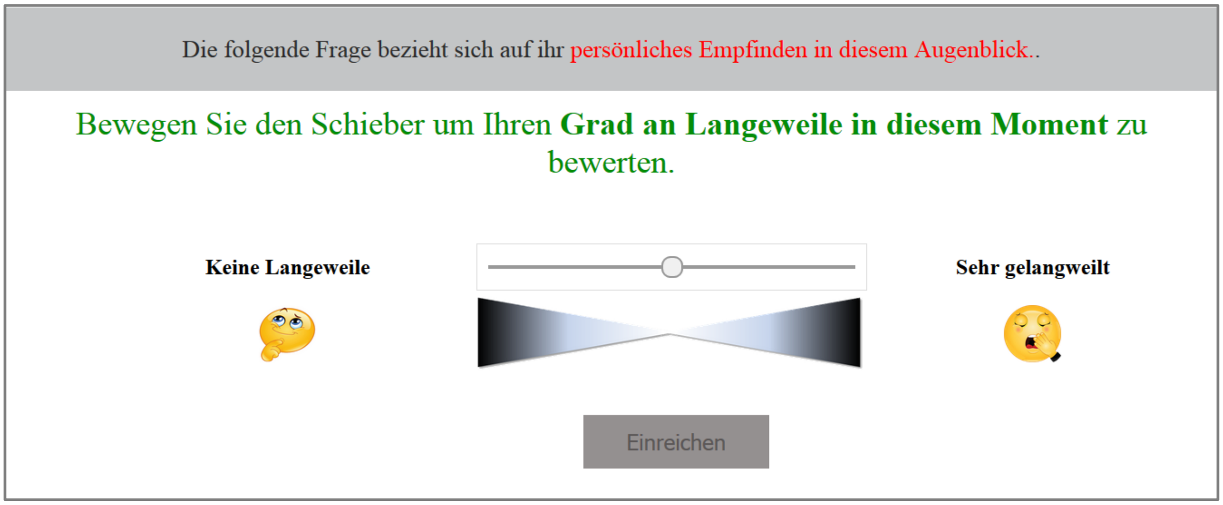

Visual analog scales for affect and arousal in German language (VAS-AA)

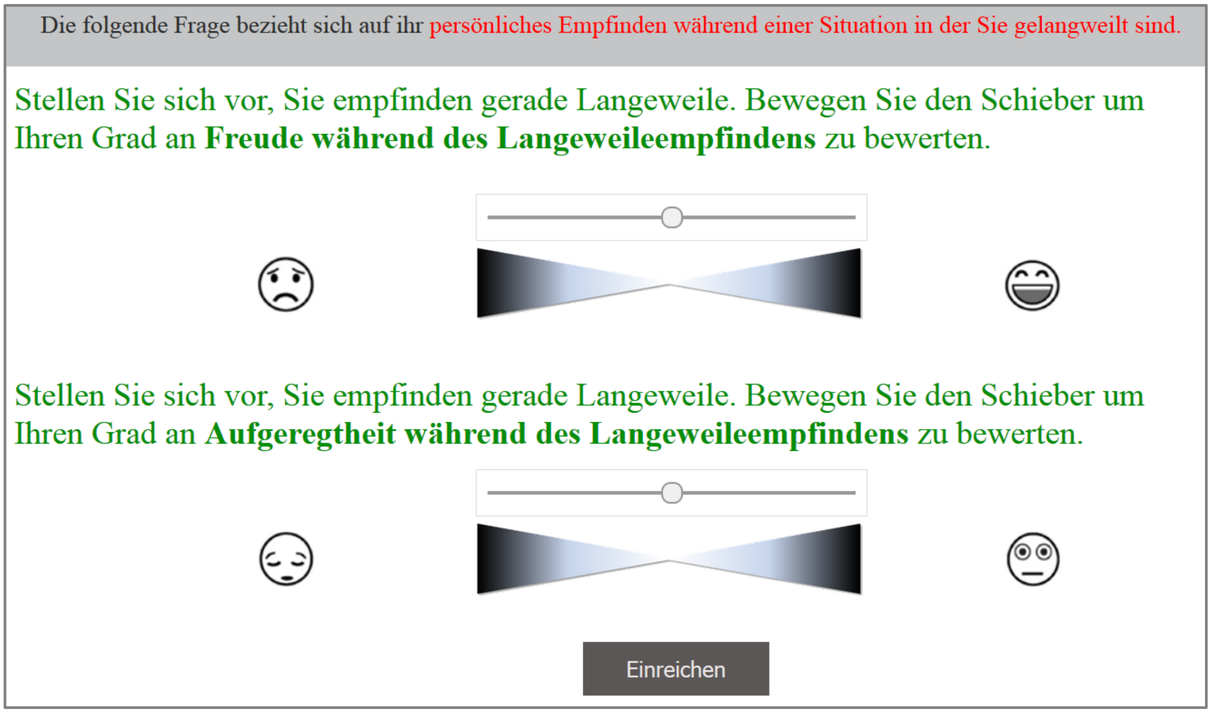

Digit span backwards task of the study

English translation of the German task version that was used in the study

Introduction: “The following task requires you to memorize some sequences of numbers and write them down in reverse order to the paper in front of you. While listening to the sequence of digits you are not allowed to use any tools or notations. Please write down the sequences in reverse order, meaning that the last named digit should be noted at the beginning of your answer. The difficulty will increase from sequence to sequence.

First, you are presented two example sequences, before moving to the task condition.”

**Test sequence I:**<br>7-2<br>Correct answer:<br>2-7	**Test sequence II:**<br>1-6-3<br>Correct answer:<br>3-6-1

(Stepwise presentation of sequences with 1s between each digit)

**Sequence I:**<br>5-1	**Sequence II:**<br>3-8
**Sequence III:**<br>4-9-3	**Sequence IV:**<br>5-2-6
**Sequence V:**<br>3-8-1-4	**Sequence VI:**<br>1-7-9-5
**Sequence VII:**<br>6-2-9-7-2	**Sequence VIII:**<br>4-8-5-2-7
**Sequence IX:**<br>7-1-5-2-8-6	**Sequence X:**<br>8-3-1-9-6-4
**Sequence XI:**<br>4-7-3-9-1-2-8	**Sequence XII:**<br>8-1-2-9-3-6-5
